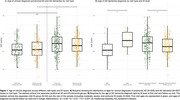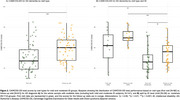# Longitudinal screening and population health strategies for early detection of Alzheimer's disease in Down syndrome

**DOI:** 10.1002/alz70858_106397

**Published:** 2025-12-26

**Authors:** Lídia Vaqué‐Alcázar, Laura Del Hoyo, Laura Videla, Bessy Benejam, Isabel Barroeta, Susana Fernandez, Íñigo Rodríguez‐Baz, Javier Arranz, José Enrique Arriola‐Infante, Lucía Maure‐Blesa, Aida Sanjuan Hernandez, Alejandra O. Morcillo‐Nieto, Alexandre Bejanin, Alberto Lleó, Maria Carmona‐Iragui, Juan Fortea

**Affiliations:** ^1^ Sant Pau Memory Unit, Hospital de la Santa Creu i Sant Pau, Institut de Recerca Sant Pau ‐ Universitat Autònoma de Barcelona, Barcelona, Spain; ^2^ Department of Medicine, Faculty of Medicine and Health Sciences, Institute of Neurosciences, University of Barcelona, Barcelona, Spain. Institut d’Investigacions Biomèdiques August Pi i Sunyer (IDIBAPS), Barcelona, Spain; ^3^ CIBERNED, Network Center for Biomedical Research in Neurodegenerative Diseases, National Institute of Health Carlos III, Madrid, Spain; ^4^ Barcelona Down Medical Center, Fundació Catalana Síndrome de Down, Barcelona, Spain

## Abstract

**Background:**

Early diagnosis of symptomatic Alzheimer's disease (AD) in individuals with Down syndrome (DS) is crucial due to its near‐universal occurrence and its significant impact on care planning and intervention strategies. This study examines the effects of key components of the screening health program implemented in Catalonia (Spain), with a particular focus on how diagnostic timing (first vs. follow‐up visit) influences the age at clinical diagnosis, cognitive status, and the moderating role of intellectual disability (ID) level in this relationship.

**Method:**

205 (50.7% female) DS with prodromal AD (pDS) and 357 (50.1% female) with AD dementia (dDS) from the Down‐Alzheimer Barcelona Neuroimaging Initiative (DABNI) cohort were split by the visit type when these diagnoses were made (first visit: N_pDS_=105, N_dDS_=219; along the follow‐up visits: N_pDS_=100, N_dDS_=138). The Cambridge Cognitive Examination for Older Adults with Down's Syndrome (CAMCOG‐DS) was used to assess the cognitive status among mild and moderate ID subjects. Parametric and non‐parametric comparisons were made for age at diagnosis and CAMCOG‐DS between patients receiving a diagnosis during the first vs. follow‐up visits.

**Result:**

Patients with pDS were younger than dDS (51.4±5.26 vs. 53.9±5.54; *t* = 2.509, *p* <0.001), with a larger difference during first visits (pDS=51.2±4.99 vs. dDS=54.1±5.72; *t* = 2.907, *p* <0.001) compared to follow‐up visits (pDS=51.5±5.55 vs. dDS=53.4±5.23; *t* = 1.950, *p* = 0.033; Figure 1A). Among individuals with mild ID, those diagnosed at their first visit were younger than those diagnosed during follow‐ups (*t* = 5.141, *p* = 0.042; Figure 1B). In contrast, for individuals with moderate, severe, or profound ID, diagnoses made at follow‐up visits tended to occur at a younger age than those made during first visits (53.01±5.18 vs. 54.4±5.66, *t* = ‐1.335, *p* = 0.035; Figure 1B). Additionally, dDS diagnosed at their first visit had lower CAMCOG‐DS total scores compared to follow‐up diagnoses (44.1±17.80 vs. 57.5±14.62; *U* = 1190.50, *p* <0.001; Figure 2A). This difference remained significant only for the moderate ID group (41.4±16.36 vs. 52.6±13.35; *z* = 3.352, *p* = 0.002; Figure 2B). No significant effects were observed for pDS.

**Conclusion:**

Longitudinal screening before AD symptoms start helps identify earlier AD clinical onset in DS depending on their ID level. Future research should address critical gaps in understanding the heterogeneity of premorbid cognition and key AD outcomes in this population.